# Night-time/daytime Protein S100B serum levels in paranoid schizophrenic patients

**DOI:** 10.1192/j.eurpsy.2023.958

**Published:** 2023-07-19

**Authors:** E. Diaz-Mesa, A. Morera-Fumero, L. Torres-Tejera, A. Crisostomo-Siverio, P. Abreu-Gonzalez, R. Zuñiga-Costa, S. Yelmo-Cruz, R. Cejas-Mendez, C. Rodriguez-Jimenez, L. Fernandez-Lopez, M. Henry-Benitez

**Affiliations:** 1 Service of Psychiatry, University Hospital of the Canaries; 2 University of La Laguna Department of Internal Medicine Dermatology and Psychiatry, School of Medicine; 3 Department of Physiology, University of La Laguna; 4 Service of Farmacology, University Hospital of the Canaries, La Laguna, Spain

## Abstract

**Introduction:**

S100B is a calcium-binding astrocyte-specific cytokine, that is considered a biomarker of neurodegeneration; which may be involved in the imbalance of the inflammatory response observed in several brain disorders, including major depression and schizophrenia. Two meta-analyses have reported higher serum levels of S100B in patients with schizophrenia respect to healthy controls.

Different studies have described circadian and seasonal variations of biological variables, such as melatonin or cortisol. It has been reported that there is not circadian rhythm of S100B blood levels in healthy subjects. However, it is not known whether there are circadian oscillations in S100B blood concentrations in patients with schizophrenia.

**Objectives:**

The aim of this study is to describe S100B serum levels in patients with schizophrenia and to analyse whether they follow a circadian rhythm.

**Methods:**

Our sample consists in 47 patients in acute phase and stabilized status. Blood samples were collected at 12:00 and 00:00 hours by venipuncture. Serum levels of Protein S100B were measured three times: at admission, discharge and three months after discharge. Protein S100B was measured by means of ELISA (Enzyme-linked immunosorbent assay) techniques.

**Results:**

**Table:** 

	12:00	24:00	P
ADMISSION	132,95±199,27	85,85±121,44	0,004
DISCHARGE	73,65±71,744	75,80±123,628	0,070
CONTROL	43,49±34,60	40,14±23,08	0,47

**Table:** 

P global	P Admission Vs. Discharge	P Admission Vs. Control	P Discharge Vs. Control
0,97

There is a significance difference between 12:00 and 24:00 at admission for the Protein S100B.However, these difference did not occur at discharge and at three months after discharge.It can be interpreted as there is a circadian rhythm of Protein S100B when the patient has got a psychotic outbreak and disappears at discharge and when is psychopathologically stable.

**Conclusions:**

With respect to our results we can hypothesize that schizophrenic patients in acute relapse present circadian S100B rhythm that is not present when the patients are clinically stable.Furthermore, the decrease of serum protein S100B levels at discharge is indicative of a reduction of the cerebral inflammation, thus it can be a biomarker of cerebral inflammation and this reduction can be the effect of the treatment. Finally, its circadianity could be a guide of this process and clinical improvement.

**Disclosure of Interest:**

None Declared

